# Biofilm antimicrobial susceptibility through an experimental evolutionary lens

**DOI:** 10.1038/s41522-022-00346-4

**Published:** 2022-10-18

**Authors:** Tom Coenye, Mona Bové, Thomas Bjarnsholt

**Affiliations:** 1 Laboratory of Pharmaceutical Microbiology, Ghent University, Ghent, Belgium; 2 Costerton Biofilm Center, University of Copenhagen, Copenhagen, Denmark

**Keywords:** Biofilms, Molecular evolution, Antimicrobials

## Abstract

Experimental evolution experiments in which bacterial populations are repeatedly exposed to an antimicrobial treatment, and examination of the genotype and phenotype of the resulting evolved bacteria, can help shed light on mechanisms behind reduced susceptibility. In this review we present an overview of why it is important to include biofilms in experimental evolution, which approaches are available to study experimental evolution in biofilms and what experimental evolution has taught us about tolerance and resistance in biofilms. Finally, we present an emerging consensus view on biofilm antimicrobial susceptibility supported by data obtained during experimental evolution studies.

## Introduction

Experimental evolution (Box 1) is the study of evolutionary processes occurring in populations in response to conditions imposed and controlled by the experimenter^1^. While the first microbial experimental evolution studies date back to the 1880's^2^, experimental evolution was introduced to bacteriology in the 1950's by Francis J. Ryan^3^ and became well-known due the long-term evolution experiment (LTEE) that was started by Richard Lenski in the 1980's and has been running for >75000 generations^4,5^. The LTEE and many other experimental evolution experiments are carried out in unstructured environments, i.e., in liquid culture with shaking, with most bacteria in a planktonic state. However, already in early evolution experiments in structured environments, marked differences in terms of evolution of fitness (Box 1) and within-population variability were observed compared to what is typically observed in planktonic cultures^6,7^.

## Biofilms are structured microenvironments—why does that matter during evolution?

Biofilms are structured microbial communities that are either attached to a surface or occur as suspended or embedded aggregates^8^. Various gradients (oxygen, nutrients, antimicrobial agents, …) are present in biofilms, resulting in the development of spatially structured niches with distinct environmental conditions^9^ and these microenvironments co-determine the outcome of biofilm-related infections, as they directly impact on bacterial growth and metabolism, as well as on the effect of antimicrobial treatment^10–13^.

Experimental evolution in general^14^ and specific aspects of experimental evolution in biofilms^15^ were recently reviewed; we refer readers to these reviews for more details. A brief summary of why biofilm populations become more diverse during evolution is presented below.

Due to their heterogeneity, biofilms contain multiple ecological niches, not all of which are used by existing genotypes; these unused niches present opportunities for novel genotypes^16^. Moreover, novel genotypes can create additional niches by altering the surrounding environment (‘niche construction’)^7,17^. Due to the spatial heterogeneity, biofilm populations can be considered as collections of independently evolving subpopulations and this population fragmentation reduces the effective population size. As the relative contribution of genetic drift (Box 1) towards diversity is higher in smaller subpopulations, spatial heterogeneity ultimately leads to more diversity^16,18^. Population fragmentation also allows fixation (Box 1) of beneficial mutations with a relatively small effect in particular subpopulations. Indeed, beneficial mutations that have a large effect are less frequent than beneficial mutations that have a small effect and the former are unlikely to appear in all subpopulations; as a consequence different beneficial mutations with a small effect are expected to occur and be maintained in different spatially separated subpopulations, leading to more diversity within the population as a whole^19^. Recent experimental work and modeling showed that in a spatially structured environment the spread of a beneficial mutation is amplified and that beneficial mutations are less likely to be lost^20^. The reason for this is that in structured environments selection can increase the frequency of a beneficial mutation in a certain subpopulation faster than the migration of this mutation to other subpopulations; as a consequence, the mutant harboring this beneficial mutation is likely to be able to migrate to novel subpopulations repeatedly, which ultimately reduces the likelihood of loss of this mutation due to genetic drift^20^. The competition between mutants harboring different beneficial mutations (clonal interference, Box 1) increases fixation times (i.e., it will take longer before a particular mutation outcompetes all others) and clonal interference is more frequent in spatially structured environments (as beneficial mutations show a slow, ‘wave-like’ spread throughout the population)^21^. As a consequence multiple beneficial mutations can co-occur in biofilms, again with a higher diversity as result^22,23^. The recent observation that in vitro evolution of *Pseudomonas aeruginosa* in conditions that are most similar to those encountered in the lung of cystic fibrosis (CF) patients (i.e., in a synthetic CF medium [SCFM]) leads to lower parallelism (i.e., more diversity) than evolution in a minimal medium, confirms the importance of the presence of spatially separated subpopulations for generating diversity^24,25^. In contrast to the minimal medium, SCFM contains mucin, which allows the creation of spatially structured subpopulations with smaller effective population sizes, making it less likely to find the same beneficial mutations in replicate populations^25^.

Finally, in homogeneous populations exposed to an antimicrobial agent, all mutations required for full resistance need to be acquired at the same time in order to avoid eradication by the uniform high concentrations of the antibiotic. However, penetration of antimicrobial agents into the biofilm can be hindered, leading to concentration gradients^9,26–29^ that can create ‘sanctuaries’, i.e., parts of the biofilm in which concentrations of antimicrobial agents are lower and that can act as ‘stepping stones’ allowing populations to acquire mutations one by one^30^. Other important aspects to consider are the increased mutation rates often observed in biofilm cells, as well as the increased rate of horizontal gene transfer (HGT) in bacterial biofilms (discussed in more detail in Box 2). It should be noted that as most experimental evolution studies are carried out with single species (see below), HGT is usually not a factor driving evolutionary changes in these studies.

Box 1. Glossary of most important terms

- **Biofilm:** structured microbial communities (attached to a surface, suspended aggregates or aggregates embedded in tissue), consisting of microorganisms embedded in a extracellular matrix composed of polysaccharides, extracellular DNA and other components^8^.
- **Clonal interference**: the competition that occurs in a population between mutants harboring different beneficial mutations^15^.
- **Experimental evolution:** the study of evolutionary processes occurring in populations established by the experimenter, in response to conditions or treatments imposed and controlled by the experimenter^14,15^.
- **Fitness**: the ability to produce more offspring (and thereby increase in frequency over time) than less-fit competitors, ideally measured in direct competition assay in which relative contribution of competitors towards future generation is assessed. Fitness is often assessed indirectly by measuring growth rate or susceptibility^14,15^.
- **Fixation**: situation in which a particular variant of a gene (mutation) is the only one remaining in the population (i.e., all others are outcompeted)^14,15^.
- **Genetic drift**: the change in frequency of a particular variant of a gene (mutation) in a population due to chance^14,15^.
- **Minimal duration for killing (MDK):** minimum time required to kill a fraction of the population; e.g., MDK_99_ and MDK_99.99_ are the times required to kill 99% and 99.99% of the cells in a population, respectively^31,32^.
- **Minimal inhibitory concentration (MIC)**: lowest concentration of an antibiotic that prevents growth of planktonic cells^31,32^.
- **Mutant selection window (MSW)**: the concentration range where the fitness of a resistant mutant is higher than the wild type^79,91^.
- **Persistence**: phenomenon in which at least two subpopulations are present in a population, one consisting of cells that are killed fast by the antibiotic and the other composed of tolerant cells that survive^48^. There is no difference in MIC and MDK_99_ between susceptible and persistent strains, but the MDK_99.99_ for the latter is substantially higher^31,32^.
- **Resistance:** Antibiotic resistant cells possess one or more mechanism that allow them to grow at antibiotic concentrations that would prevent the growth of susceptible bacteria. Examples include reduced uptake and increased efflux of antibiotics, modification of the target, and (enzymatic) inactivation of the antibiotic^31,32,125^.
- **Tolerance:** population-level phenomenon allowing a population to survive exposure to an antibiotic (at levels above the MIC) without involvement of a resistance mechanism. Tolerant cells are often non- or slowly growing and can regrow after the antibiotic is removed. There is no difference in MIC between a tolerant and a susceptible strain, but the MDK_99_ is substantially higher for a tolerant than for a susceptible strain^31,32,125^.

Box 2. Evidence for increased mutation rates and horizontal gene transfer (HGT) in bacterial biofilms

- The rate of point mutations in bacteria varies between 10^-10^ –10^-9^ per bp per replication^93,133^ although mutation rates may be 100 to 1000-fold higher in hypermutators (strains with elevated mutation frequency due to mutations in DNA mismatch repair genes^134–136^).
- Mutation rates have been compared between planktonic cultures and biofilms for several organisms (including *Pseudomonas aeruginosa, Escherichia coli, Streptococcus pneumoniae, Staphylococcus aureus* and *Staphylococcus epidermidis*) and were found to be substantially (4 to >100-fold) higher in biofilms^104,116,118,137^.
- However, chemical gradients in biofilms lead to physiological heterogeneity^9^, which is also reflected in marked differences in gene expression and growth rates, with biofilms often containing a considerable fraction of slowly growing and non-dividing cells^138,139^. This complicates the direct comparison of mutation rates (typically expressed per bp per replication) between heterogeneous biofilms and well-mixed planktonic cultures^114^.
- Increased mutation rates could be linked to oxidative stress, as in biofilm-grown *P. aeruginosa* PAO1 the expression of genes coding for enzymes conferring protection against oxidative DNA damage was downregulated, e.g., the expression of *katA* (coding for the major catalase responsible for converting hydrogen peroxide to oxygen and water) was 7.7-fold downregulated^137^. In line with this, in other studies the production of hydrogen peroxide was found to be important for increased mutation rates in streptococci and staphylococci^116,118^.
- The relevance of oxidative stress is confirmed by observations linking double-stranded DNA breaks caused by endogenous oxidative stress and the subsequent repair of these breaks by mechanisms that introduce mutations, with biofilm adaptation^117,122^.
- Biofilms also provide ample opportunity for HGT, and its rates are typically higher in biofilms than in planktonic cultures^140,141^, although they may be affected by the spatial separation of donor and recipients^142^, the type of plasmid^143^, the sequence and length of the specific DNA fragment^144^, and the overall biofilm architecture (including presence of exopolysaccharides in the matrix)^145^.

## Recent insights into development of reduced antimicrobial susceptibility from experimental evolution with planktonic populations

### Antimicrobial resistance

Antimicrobial resistance is quantified by the minimal inhibitory concentration (MIC, Box 1)^31^. The ability of resistant organisms to grow at concentrations above the MIC for susceptible organisms is linked to the presence of one or more resistance mechanisms^32^ and evolutionary trajectories towards a resistant phenotype can be complex^33^. Experimental evolution in which cultures are serially passaged (in the presence of a constant or gradually increasing concentration of an antibiotic), combined with whole-genome sequencing (WGS) can be used to identify resistance of and trajectories towards resistance^34^. For example, serial passaging of *Escherichia coli* in the presence of carbapenems allowed identification of several previously unknown carbapenem resistance mechanisms, including mutations in *mrdA* (coding for PBP2) and *ftsI* (coding for PBP3), both targets of carbapenems, as well as mutations in *acrB* (coding for the inner membrane associated part of the AcrAB-TolC efflux pump)^35^. Experimentally evolving *E. coli* in the presence of chloramphenicol induced mutations in the DNA binding region of *marR*, which can upregulate the AcrAB-TolC efflux pump, as well as mutations in *acrB* and *acrR* (interruption of *acrR* leads to upregulation of *acrAB*)^36^. In *Streptococcus pneumoniae*, experimental evolution in the presence of increasing concentrations of moxifloxacin and levofloxacin led to the identification of novel mutations in *gyrB*, that in combination with mutations in *gyrA* and *parC* lead to high-level fluoroquinolone resistance^37^. In an experimental evolution study with *P. aeruginosa*, both expected (e.g., mutations leading to AmpC overproduction after evolution in the presence of ceftazidime, mutations in *oprD* leading to inactivation of the porin after evolution in the presence of meropenem) and novel (e.g., gain-of-function mutations leading to the structural modification of AmpC after evolution in the presence of ceftazidime, novel mutations in *gyrA* after evolution in the presence of ciprofloxacin) resistance mechanisms were identified^38^. There is a growing body of evidence that metabolic adaptations and reduced antimicrobial susceptibility go hand in hand^39,40^, and several experimental evolution studies with planktonic *E. coli* populations have recently confirmed this. When *E. coli* is grown in a minimal medium with glucose (supporting rapid growth with respiration or fermentation) or acetate (supporting slower growth with respiration only), resistance develops much faster on glucose, confirming that environmental conditions constrain the rate of resistance development^41^. Most changes observed involve metabolic processes that are not directly affected by the antibiotic treatment. For example, cultures evolved in the presence of glucose and chloramphenicol consume more glucose, secrete more acetate and show reduced oxygen uptake compared to wild type *E. coli* and *E. coli* adapted in the presence of glucose only, indicating a metabolic switch from respiration to fermentation. This switch is linked to overexpression of the AcrAB efflux pump (required for chloramphenicol resistance) and membrane proteome remodeling, due to competition for space between efflux pump and proteins involved in oxidative phosphorylation^41^. Further evidence for the role of metabolic changes in the development of antimicrobial resistance comes from experimental evolution of planktonic *E. coli* using both a conventional experimental evolution protocol and a ‘metabolic evolution protocol’ designed to ensure equivalent selection dynamics for all conditions, by exposing bacteria to antibiotics at different temperatures (i.e., at increasingly heightened metabolic states)^42^. Evolution under the conventional settings leads to more slowly growing populations with an increased MIC; mutations frequently found in these populations are in genes linked to known resistance mechanisms. However, a subset of clones acquires mutations in other genes, including genes related to central metabolism (TCA cycle, electron transport). Populations obtained at the end of the ‘metabolic evolution’ experiment exhibit increased survival in killing assays compared to the ancestral wild type strain, without reduction in exponential growth rate or increase in lag time (ruling out tolerance [Box 1] due to slow growth). Engineering mutants in six metabolic genes further confirmed the relevance of these mutations as in all mutants the MIC to at least one antibiotic was increased. The mechanism by which these mutations provide resistance vary, but for at least one of them (*sucA*, encoding the TCA cycle enzyme 2-oxoglutarate decarboxylase) the mutation provides resistance by lowering basal respiration and thereby preventing antibiotic-mediated induction of TCA cycle activity, a mechanism previously observed in different organisms^43–45^. 39% of coding sequence mutations identified in these evolution experiments can also be found in sequenced *E. coli* genomes; moreover, several mutations in metabolic genes are abundantly present in these genomes and some are statistically enriched in clinical *E. coli* isolates, suggesting they are relevant in vivo^42^.

### Antimicrobial tolerance and persistence

Reduced susceptibility to antibiotics is not only due to resistance, as also tolerance and persistence (Box 1) play important roles^31,32,46–48^. Tolerant cells survive exposure to antibiotics without carrying conventional resistance mechanisms and will resume growth after removal of the antibiotic^32^. The factors that lead to tolerance can be genetic (e.g., mutations leading to increased lag time^49,50^) or environmental (e.g., production of a protective biofilm matrix^51,52^, slow growth due to microenvironmental conditions^53,54^). Tolerance and persistence can both be quantified by the minimum duration for killing (MDK, Box 1); in addition, persistence is typically characterized by the presence of a biphasic killing curve^31,32^. Cyclic exposure of planktonic *E. coli* cultures to ampicillin led to an increase of the MDK and this increase was due to an extended single-cell lag time; no changes in MIC were observed, ruling out resistance^49^. When planktonic populations of various ESKAPE pathogens were cycled between exposure to aminoglycosides and regrowth, a 37 to 213-fold increase in number of persister cells was observed upon treatment of evolved clones compared to the start culture, again without an increase in MIC^55^. WGS of evolved high-persistence clones showed that this phenotype could be attributed to a single mutation in either *oppB, gadC* or *nuoN*, genes not previously implicated in persistence^56^.

### Tolerance and persistence as ‘stepping stones’ towards resistance

Experimental evolution has shown that the development of tolerance and persistence can be ‘stepping stones’ towards the development of resistance. When planktonic *E. coli* cultures were evolved in the presence of ampicillin, mutations in the promotor region of *ampC*, encoding a β-lactamase, increased the MIC after 7–17 cycles, while delayed growth was already observed after 3–4 cycles, i.e., development of tolerance preceded that of resistance^57^. WGS of the first resistant clones showed that all carried additional mutations of which some had previously been identified as increasing tolerance by increasing the lag time^49^; additional sequencing revealed that the same tolerance mutations had been present prior to the appearance of the *ampC* resistance mutations. As mutations in several genes can lead to tolerance, the target size for mutations leading to tolerance is larger than that for resistance (*ampC* being the only target); as a consequence tolerance mutations occur more frequently and can be detected earlier. Starting evolution experiments from wild type strains and from strains that had already developed tolerance demonstrated that resistance mutations established faster in tolerant clones The survival advantage conferred by resistance mutations upon exposure to high ampicillin concentrations is comparable to that of the tolerance mutations (as *ampC* resistance mutations only result in partial resistance). As a result, tolerance mutations start dominating the population after a few cycles and the presence of these mutations reduces the probability of loss of resistance mutations during antibiotic treatment^57^. Similar observations were made for *P. aeruginosa*: upon sequential exposure, *P. aeruginosa* rapidly adapts to high concentrations of tobramycin with a stepwise increase in survival rate and after 7-8 cycles all evolved lineages had reached MICs substantially higher than the ancestral strain^58^. WGS showed that alleles occurred and reached fixation in a specific order, with mutations in genes involved in respiration and energy metabolism (leading to tolerance) typically preceding the acquisition of resistance mutations, and periodically exposing *P. aeruginosa* wild type, and mutants with various levels of tolerance, to tobramycin confirmed that the rates of resistance acquisition were similar in all groups but that tolerant lineages were more likely to survive the initial selection. This suggests that bacterial populations with high tolerance have a better chance to develop resistance than populations with low or no tolerance^58^. Finally, several studies have pointed towards a link between persistence and the likelihood of developing resistance, e.g., in *Mycobacterium tuberculosis*^59^, *Pseudomonas* spp.^60^ and *E. coli*^61^.

## Tools to study experimental evolution in biofilms

**Fig. 1:**
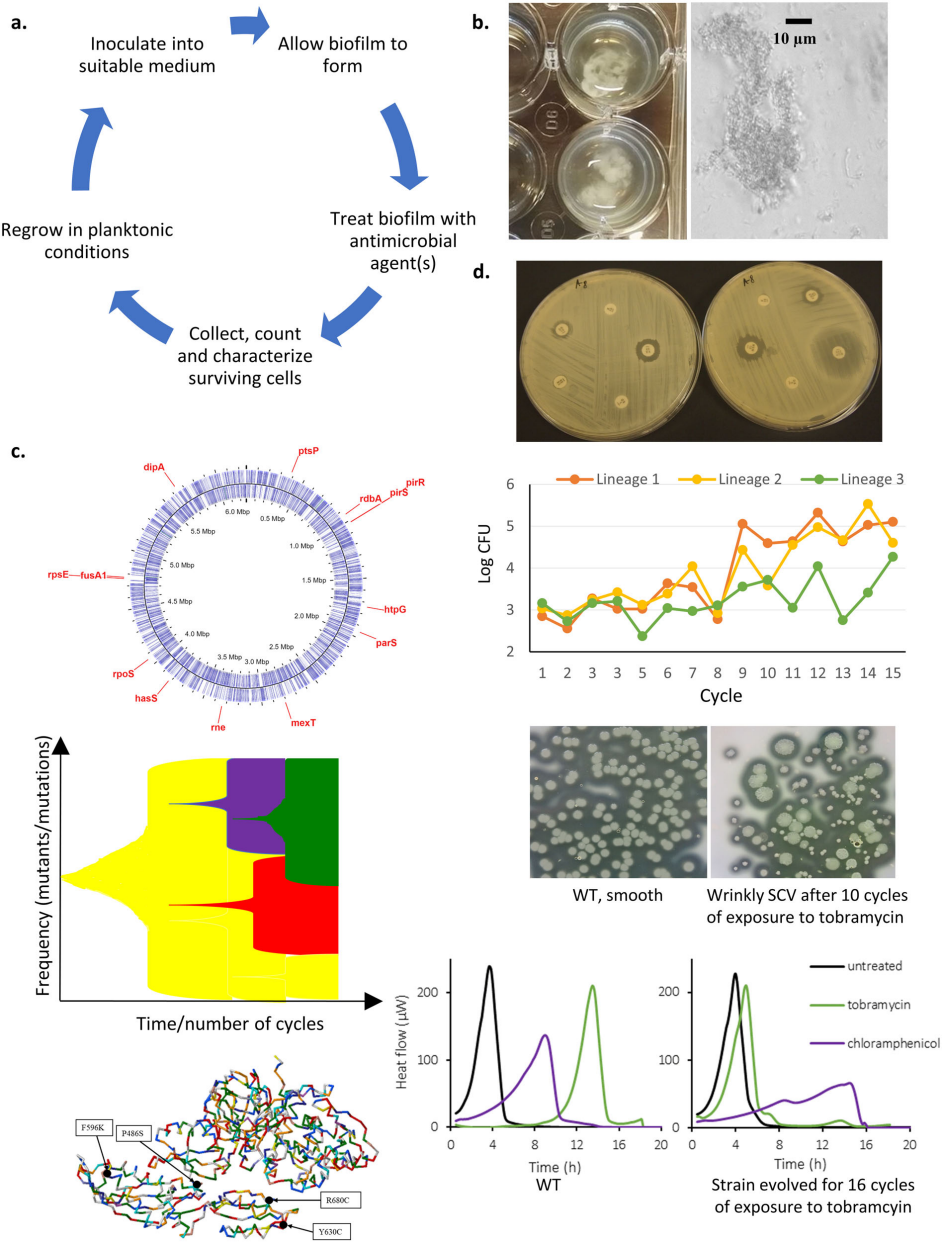
Experimental evolution in biofilms. **a** Schematic overview of the general set-up of experimental evolution experiments involving antimicrobial treatment of biofilms. **b** *P. aeruginosa* readily forms aggregates in SCFM2, making this a suitable growth medium to study evolution in a relevant microenvironment. **c** Based on whole-genome sequence analysis (mutations occurring in *P. aeruginosa* PAO1 after repeated exposure to furanone C-30 shown as example^89^), frequency of mutations can be calculated and effect of mutations on protein function (*fusA1* shown as example^89^) can be estimated. **d** Phenotypic characterization typically starts with determining antimicrobial susceptibility (illustrated here with disk diffusion) and the number of CFU (number of CFU in three replicate *B. cenocepacia* populations after repeated cycles of exposure to tobramycin are shown as example^70^). Experimental evolution in biofilms frequently leads to the occurrence of small colony variants (SCV) (*P. aeruginosa* AA2 shown as example, picture courtesy of Dr. A. Sass). Finally, changes in metabolism occur during evolution and can be measured using for example microcalorimetry; metabolic activity after treatment of WT *P. aeruginosa* PAO1 (left) or the same strain evolved in the presence of tobramycin (right) is shown as example.

For a detailed overview of available biofilm methods, we refer to recent reviews^62–65^. Importantly, while the general set-up in most evolution experiments is similar (with repeating cycles of growth, treatment and transfer to a new environment) (Fig. 1a), the model used can profoundly impact the outcome of the experiment and not every in vitro model will mimic evolution in vivo, e.g., many models use surfaces and growth media that are poorly reflective of the in vivo conditions^15^.

### Static and dynamic model systems

In static systems, biofilms will be grown and treated, and subsequently cells will be collected to initiate a new cycle. In dynamic systems a biofilm is continuously grown and treated, without being disrupted.

Biofilms formed on plastic or glass beads are frequently used to study evolution^17,66–72^. The bead model was originally developed for the selection for daily adherence to, and dispersal from, a bead by *Burkholderia cenocepacia*^22^. In this set-up, a bead is incubated together with bacteria that will attach to the bead to form a biofilm. The bead that contains the biofilm is then transferred to another recipient tube containing a new empty bead and fresh medium, which will allow colonization of the new bead, without disturbing the biofilm. Variants of this model that are more tailored towards studying the responses to antimicrobial treatment have also been developed^70,73^. Despite the fact that it only allows studying surface-attached biofilms, the ease of use and the compatibility with different organisms and growth media make this an attractive model.

Colony biofilms can be formed on membrane filters that are inoculated with the test organism and are subsequently placed on a suitable growth medium^74,75^. Nutrients will diffuse through the membrane, and the bacteria will form a biofilm on the filter membrane. During the experiment the filter can be moved to another agar plate and this way biofilms can easily be exposed to antimicrobial agents. At the end of a cycle, the bacterial cells are detached from the membrane and the resulting suspension can be used to inoculate a new filter membrane.

The Calgary biofilm device consists of a 96-well plate and a lid with pegs, which are each submerged in a well and support biofilm formation; this device was originally developed to determine the minimal biofilm eradication concentration^76^. During experimental evolution, the lid with pegs can easily be transferred to a new 96-well plate and biofilms can be dispersed from the pegs via sonication. The resulting cell suspensions can then be used to start biofilm formation on pegs on a new lid^77^. A conceptually-similar system (FlexiPeg) was recently developed and used to study competition and fitness in biofilms^78,79^.

In dynamic model systems biofilms are grown on a surface while nutrient and waste products are continuously added and removed, respectively. While technically more demanding, an advantage of these systems is that the biofilm does not have to be dispersed in between different treatment cycles. Examples include acrylic flow cells with a glass surface^80^, Sartorius bioreactors^81–83^ and various microfluidic devices^84–86^.

While many studies make use of standard growth media (e.g., LB broth), it is possible to more closely mimic the in vivo environment by using validated in vivo-like media. This includes various artificial CF sputum media^87^ in which suspended bacterial biofilm aggregates rapidly form (Fig. 1b) and which were used to study development of ciprofloxacin resistance^88^ as well as resistance to the combination tobramycin/furanone C-30^89^ in *P. aeruginosa* biofilms.

### Selective pressure during experimental evolution

The choice of the appropriate selective pressure is an important decision in evolution experiments and can profoundly affect the outcome of the experiment.

When studying mechanisms of adaptation, the antibiotic concentration should be sufficiently high to have an effect on the bacteria, but cannot be too high in order to allow survival of a sufficient number of bacteria to initiate a next cycle of the experiment. There is no information on which concentration range constitutes the mutant selection window (MSW, Box 1) for biofilms and various aspects of biofilm biology likely affect this window^90,91^. While it has been predicted that biofilm growth leads to shifts and distortions of the MSW^91^, recent work with *E. coli* showed that minimal selective concentration values (for five different antibiotics) did not differ between planktonic cultures and biofilms^79^. Due to the uncertainty concerning the biofilm MSW, antibiotic concentrations are often selected based on the MIC^70^ or on the minimal biofilm inhibitory concentration^74^. An alternative strategy is using antimicrobial concentrations that are achievable in vivo, e.g., in sputum from CF patients after inhalation therapy^75^. Selection strength can profoundly influence evolutionary trajectories, e.g., sublethal concentrations of tigecycline select for *P. aeruginosa* mutants with lower tigecycline MICs and higher MICs to other antibiotics than mutants selected under lethal concentrations^92^. In general, in vitro evolution in the presence of a mild selective pressure leads to a more diverse population, while exposure to a high selective pressure eliminates bacteria with intermediate susceptibility, and will only result in the detection of those mutations that have the strongest effect^93^. The concentration of an antibiotic at the site of infection depends on the mode of administration, and while high concentrations may be achievable with inhalation therapy or topical application, the antibiotic concentration at the infection site will often be substantially lower when antibiotics are systemically administered^94,95^. In addition, biofilms can be considered as independent pharmacological microcompartments^96,97^ and diffusion limitations often lead to formation of gradients of antibiotic concentrations in a biofilm^26–29,98^.

Evolutionary trajectories will differ between antibiotics belonging to different classes, e.g., when *P. aeruginosa* was evolved in the presence of sublethal tobramycin or tigecycline concentrations, mutants were selected at sublethal concentrations of tigecycline only^92^. While these trajectories will depend on the mode of action of the antimicrobial agents, different classes of bactericidal antibiotics also have common aspects, including that they mostly inhibit biosynthesis of macromolecules (DNA, proteins, peptidoglycan) and induce changes in metabolism that promote the formation of reactive oxygen species^99,100^. In addition, the activity of some antibiotics strongly depends on microbial metabolism while other antibiotics only weakly depend on metabolism for their killing activity^101^; tolerance will quickly develop towards the former group of antibiotics, but not the latter^102^.

Finally, the treatment regime can have an influence on the evolutionary trajectory that is followed. The concentration of the antimicrobial agent can be kept constant during the course of the evolution experiment^70,75^ or bacteria can be exposed to gradually increasing antimicrobial concentrations^82,85^, and exposure can be continuous^69,74,75,82,85^ or intermittent^70,77,103,104^. Regrowth of the biofilm after each treatment cycle ensures that biofilms with similar cell densities are studied throughout the experiment, and the regrowth phase can mimic the decrease of antibiotic concentration in between two treatments. In addition, continuous exposure may impose growth-dependent selection which can be avoided by separating treatments by rounds of antibiotic-free growth^42^.

### Studying evolution in multispecies communities and in vivo

Experimental evolution of antimicrobial susceptibility has not yet extensively been studied in more complex settings although several studies demonstrate that evolution experiments with polymicrobial biofilms are feasible; examples include a dual species biofilm (*Acinetobacter* sp. + *Pseudomonas putida*) evolved on benzyl alcohol^105^, evolution of *P. aeruginosa* in the presence of *Staphylococcus aureus*^106^ or members of the CF microbiome^107^, and a 34-species model bacterial community repeatedly exposed to streptomycin^108^. Examples of in vivo studies include serial propagation of *S. pneumoniae* by repeated murine nasal colonization^109^ and adaptation of *Shewanella oneidensis* to life in the intestines of larval zebrafish^110^. Recently a *Caenorhabditis elegans* infection model was used to show that repeated exposure of *B. cenocepacia* to anti-virulence compound FR900098, an inhibitor of the non-mevalonate pathway, did not lead to changes in susceptibility to this compound^111^.

### Interstrain variability and selection of isolates

When selecting isolates for experimental evolution studies and when analyzing the results, interstrain variability should be taken into account. For example, based on in vitro biofilm morphology and transcriptional profiles, clinical *P. aeruginosa* isolates can be grouped in different clusters and strains in different clusters share only a restricted core biofilm transcriptional profile; these differences appear shaped by the genetic background of the individual strains rather than the maturation status of the biofilm^112^. Also tolerance is to a large extent determined by the individual strain background and this strain-dependent tolerance is also antibiotic-dependent, with cross-tolerance of clinical *P. aeruginosa* isolates observed for ciprofloxacin and tobramycin, but not colistin^113^. This interstrain variability may have a profound impact on evolutionary trajectories during experimental evolution and will likely complicate the elucidation of the contribution of specific tolerance and resistance mechanisms to reduced susceptibility. At the same time it highlights the versatility of bacterial pathogens to come up with parallel solutions.

## What has experimental evolution in the presence of antibiotics taught us about tolerance and resistance in microbial biofilms?

Changes in antimicrobial susceptibility are not only observed when populations are evolved in the presence of an antimicrobial agent, but have also been observed in some experimental evolution studies in which biofilms are evolved in the absence of antibiotics (e.g., in *E. coli*^114^ and *P. aeruginosa*^74,115^). These changes are likely the result of higher mutation rates in biofilms (Box 2) and, combined with a range of other mechanisms involved in reduced susceptibility^12,39^, the resulting diversity helps survival of the population (‘insurance hypothesis’)^15,116–118^. In the next section we however focus on experimental evolution studies investigating changes in biofilm antimicrobial susceptibility occurring during exposure to antibiotics.

### P. aeruginosa

An non-exhaustive overview of genes mutated in *P. aeruginosa* biofilms during experimental evolution in the presence of antibiotics is shown in Table 1.

**Table 1:** Non-exhaustive overview of genes mutated in *P. aeruginosa* and *A. baumannii* biofilms during experimental evolution in the presence of antibiotics.

Species	Reference	Model system, strain	Antibiotic	Genes mutated in treated	
				Biofilms	Planktonic cultures
*P. aeruginosa*	^88^	CF medium, PA14	Ciprofloxacin	Target: *gyrA, gyrB*	–
				Efflux: *nfxB*	
				Others: PA14_32420 (oxidoreductase), *orfN* (glycosyl transferase)	
	^74^	Colony biofilm, PAO1	Ciprofloxacin	Efflux: *mexR, oprM, nfxB, mexS*	Efflux: *mexR, nalC, nalD*
				Metabolism: *nuoJ*, PA1054, PA1252	Target: *gyrA, gyrB*
	^121^	Colony biofilm, PAO1	Ciprofloxacin	Target: *gyrA, gyrB, parC*	Target: *gyrA, gyrB*
				Efflux: *mexR, nfxB*	Efflux: *mexR, nalC*
				Metabolism: *sdhA*, many genes in Arg and	Metabolism: *sdhA*
				polyamine transport/metabolism	
				RNA polymerase: *rpoS, rpoN*, PA1300	RNA polymerase: PA1300
	^73^	Beads, PA14	Tobramycin	Target: *fusA1*	Target: *fusA1*
				O-antigen: *orfKHLN*	Metabolism: *ptsP*
*A. baumannii*	^124^	Flow system, AB5075	Ciprofloxacin	Target: *smpB*	-
				Efflux: *adeS*	
				Capsular polysaccharide: ABUW_3824,	
				ABUW_3825	
	^69^	Beads, ATCC 17978	Ciprofloxacin	Efflux: *adeL, adeS*	Efflux: *adeN*
					Target: *gyrA, parC*
	^73^	Beads, ATCC 17978	Tobramycin	Target: *fusA1*	Target: *fusA1*
				Metabolism: *ptsP, cyoA*	Metabolism: *ptsP*

In *P. aeruginosa* PAO1 colony biofilms formed on polycarbonate membranes, exposure to subinhibitory concentrations of ciprofloxacin rapidly induced reduced susceptibility to this antibiotic^74^. After 7 passages, the size of the resistant subpopulations was significantly larger in biofilms than in planktonic populations and the mean MIC of ciprofloxacin towards selected colonies derived from ciprofloxacin-evolved biofilms increased significantly during the experimental evolution; the latter was not observed for colonies derived from planktonic cultures (although the clones with the highest MIC values were derived from planktonic cultures)^74^. Both the number of mutations and the mutational spectrum differed between evolved populations: a significantly higher number of nonsynonymous mutations was observed in the ciprofloxacin-evolved populations, transitions were more frequent in planktonic populations, and transversion and indels were more frequent in biofilms (the latter potentially linked to higher activity of insertion sequences under oxygen-limited conditions^119,120^). Mutations in *mexR* (regulator of efflux pump MexAB-OprM), *nfxB* (MexCD-OprJ) and *mexS* (MexEF-OprN) were frequent in biofilms evolved in the presence of ciprofloxacin, while mutations in *nalC* and *nalD* (regulators of MexAB-OprM) as well as in *gyrA* and *gyrB* were frequently found in ciprofloxacin-evolved planktonic populations. In addition, low-frequency mutations in genes related to metabolism were found in several biofilms evolved in the presence of ciprofloxacin; mutated genes include PA1252 (malate dehydrogenase), *nuoJ* and PA1054 (NADH dehydrogenase)^74^. Additional mutations linked to metabolism identified in ciprofloxacin-exposed biofilms include mutations in genes related to the TCA cycle (e.g., *sdhA*) and polyamine and arginine metabolism and transport (e.g., *argS*), as well as in genes encoding various sigma factors (including *rpoN* and *rpoS*)^121^. The latter mutations might help explain the prolonged lag phase and increased doubling times observed in ciprofloxacin-resistant clones recovered from evolved biofilms. Overall these data suggest that biofilm-grown *P. aeruginosa* cells exposed to subinhibitory ciprofloxacin concentrations more frequently carry mutations leading to low-level resistance, which could in turn accelerate the stepwise development of ciprofloxacin resistance in vivo^74^. Interestingly, under the same experimental conditions, lack of the major *P. aeruginosa* catalase KatA increased the fraction of the ciprofloxacin-resistant population in biofilms and more mutations were observed in evolved Δ*katA* biofilms^75^, again highlighting the role oxidative stress can play in generating diversity in biofilms^122^. Nevertheless, the observation that ciprofloxacin-resistant mutants also appear after evolving biofilms under anaerobic conditions demonstrates oxidative stress is not the only mechanism^75^.

Using a bead based model, *P. aeruginosa* PA14 biofilms were evolved in the absence or presence of increasing tobramycin concentrations. In the biofilm evolved in the presence of tobramycin, MIC values increased 16-fold and at the end of the experiment all tobramycin-exposed biofilms had acquired mutations in *fusA1* (encoding elongation factor G)^73^. While *fusA1* mutations also occurred in tobramycin-exposed planktonic populations, they dominated in all final biofilm populations while in planktonically evolved populations their frequencies were more variable. Investigating evolved mutant clones revealed that *fusA1* mutations alone lead to 2 to 4-fold increase of tobramycin MIC and at least a 6-fold increase in the tobramycin concentration in which biofilms survived. *P. aeruginosa* biofilm populations also frequently acquired mutations in the *orfKHLN* genes (encoding O antigen biosynthesis enzymes) and mutants with mutations in *orfN* and *fusA1* mutations were more resistant than mutants with mutations in *fusA1* alone.

Finally, experimental evolution in *P. aeruginosa* biofilms and planktonic cultures was recently used to identify mechanism of resistance towards the engineered cationic antimicrobial peptide WLBU2^123^. WGS revealed that surviving populations had minimum two mutations among three key functional categories, i.e., LPS modification (*pmrB*), O-antigen biosynthesis (*orfN*) and biofilm formation (*wspF* and *morA*). While *pmrB* and *orfN* are known to be involved in resistance to cationic peptides, the occurrence of mutations in genes of the *wsp* pathway (selected both in biofilms and planktonic cultures) was more unexpected. Resistant clones with *wsp* mutations showed more aggregation, suggesting that increased aggregate and/or biofilm formation itself could contribute to WLBU2 resistance^123^.

### Acinetobacter baumannii

An non-exhaustive overview of genes mutated in *A. baumannii* biofilms during experimental evolution in the presence of antibiotics is shown in Table 1.

Using a flow model in which *A. baumannii* biofilms are formed in plastic tubes attached to a peristaltic pump, the effect of exposure to ciprofloxacin (0.5 x MIC) and tetracycline (0.25 x MIC) was investigated^124^. Cells dispersed from biofilms exposed to antibiotics had a higher MIC with 93% of isolates from ciprofloxacin-treated biofilms showed increased resistance towards ciprofloxacin and 53% isolates from tetracycline-treated biofilms showed increased resistance towards tetracycline; 80% of isolates from ciprofloxacin-treated biofilms also showed increased resistance to tetracycline but cross-resistance was not observed in isolates from tetracycline-treated biofilms. Mutations selected in cells from ciprofloxacin-treated biofilms could often directly be linked to resistance, e.g., mutations in *smpB* (the deletion of which leads to increased resistance to fluoroquinolones, possibly due to a preventive effect on chromosome fragmentation) and in *adeS* (leading to overexpression of the AdeABC efflux system)^124^. Mutations in two genes belonging to the K-locus (production of capsular polysaccharide) were found in samples exposed to either antibiotic and these mutations were often linked with antibiotic resistance phenotypes. Several genes were commonly mutated in isolates from tetracycline-treated biofilms; these mutations often positively correlated with increased biofilm formation rather than increased resistance to tetracycline and include a large 8706 bp deletion in a region encoding proteins involved in regulating c-di-GMP levels^124^.

The above-mentioned bead model has also been used to study evolution of *A. baumannii* biofilms in the presence of ciprofloxacin^69^ or tobramycin^73^. Comparison of planktonic cultures and biofilms exposed to increasing concentration of ciprofloxacin showed that high-level resistance quickly developed in planktonic cultures (~160-fold increase in MIC) while mutants with low levels of resistance (~6-fold increase in MIC) occurred in biofilms^69^. Mutations disrupting the repressors *adeL* (regulator of the AdeFGH efflux pump) or *adeN* (regulator of the AdeIJK efflux pump) dominate in biofilm and planktonic clones, respectively, suggesting the presence of lifestyle-specific efflux systems, as previously identified in other organisms^125^. Interestingly, mutations in *adeS* (regulator of the AdeABC efflux pump) appeared in exposed biofilms, but were subsequently outcompeted by *adeL* mutations, something not observed in another study with *A. baumannii*^124^. While a couple of mutations quickly reached fixation in planktonic populations (including a single high frequency mutation in *gyrA* in genetic backgrounds containing an *adeN* mutation) more diversity was maintained in biofilms. *A. baumannii* biofilms propagated under tobramycin selection demonstrated an 8 to 32-fold increase in MIC and also in this species mutations in *fusA1* occurred in all replicate populations exposed to tobramycin^73^. In contrast to *P. aeruginosa* biofilms, tobramycin-treated *A. baumannii* biofilms quickly accumulated mutations in *ptsP* (encoding phosphoenolpyruvate phosphotransferase), and *fusA1* and *ptsP* mutations reached similar frequencies in treated biofilm and planktonic populations. Evolved mutant clones with only a mutation in *fusA1* showed a 4-fold increase in MIC, while *fusA1 ptsP* double mutants showed an 8-fold increase. In contrast to *fusA1* (which is an essential gene), *ptsP* mutations are likely loss-of-function mutations as they are indels that lead to a frameshift. In addition, six mutations in *cyoAB* (coding for two subunits of cytochrome bo_3_ ubiquinol oxidase involved in the electron transport chain) only occurred in biofilms; these mutations were however outcompeted by the *fusA1 ptsP* genotype at higher tobramycin concentrations^73^.

### E. coli and Salmonella

*E. coli* biofilms grown in flow cells in the presence of rifampicin or kanamycin were used to address the question how growth in a biofilm can protect resistant cells from being outcompeted by fitter non-resistant cells in the absence of antibiotics^80^. Because of physical constraints and biofilm heterogeneity, it can reasonably be assumed that individual cells only have to compete with a subset of other cells^15^, while in unstructured planktonic populations cells would experience global competition in which they have to compete against all other cells^126^. The inoculum already contained low levels of kanamycin and rifampicin‐resistant mutants and during biofilm formation in the absence of antibiotics, their number increased ~45-fold. Treatment with rifampicin led to fixation of rifampicin resistance (i.e., the entire population became resistant), while kanamycin treatment resulted in a population with 52% resistant cells. When the treatment was stopped, the fraction of resistant cells did not change, but when biofilm cells were transferred to planktonic cultures, kanamycin (but not rifampicin) resistance gradually returned to the original low levels^80^. This study shows that resistance in biofilms can be the result of *de novo* mutations, but can also be due to selection of pre-existing mutants that are less fit outside the biofilm environment. *E. coli* biofilms that are grown on silicone disks and are intermittently exposed to high (5 x MIC) and very high (80 x MIC) concentrations of amikacin experience a strong drop in surviving cell number after the first treatment, but the number of surviving cells quickly increases (to ~100% survival for exposure to 5 x MIC and ~1% survival for exposure to 80 x MIC)^104^. In planktonic cultures, the decrease after the first treatment is more pronounced and only ~0.1% of the cells ultimately survive exposure to 5 x MIC (no survivors are observed after three cycles with exposure to 80 x MIC). This increased survival in biofilms is associated with a rapid MIC increase in treated biofilms, while the MIC increase in planktonic cultures is much lower. Mutations in *sbmA* (coding for an inner membrane peptide transporter previously associated to increased *E. coli* resistance to aminoglycosides) were found in all treated biofilm populations and two out of three treated planktonic populations, but not in non-treated controls; five out of six evolved biofilm populations had multiple *sbmA* mutations, suggesting clonal interference. Mutations in *fusA* were selected in several intermediate biofilm populations and at the end of the experiment in one biofilm population; no *fusA* mutations were selected in planktonic cultures. *fusA* and *sbmA* can coexist in biofilm populations but *fusA* mutations appear sooner (or the latest at the same time) than *sbmA* mutations^104^. In the absence of antibiotics *fusA* mutants have a lower fitness than *sbmA* mutants, suggesting the former were counter-selected in the periods between treatment in planktonic cultures while they were maintained in biofilms. Loss-of-function mutations in the *sbmA* gene lead to a moderate increase of the MIC (from 16 to 24 µg/ml), while *fusA* mutations lead to MIC values of 48 µg/ml. Highest MIC values (128 µg/ml) were observed in clones that harbored a mutation in *fusA* combined with a loss-of-function mutation in *sbmA* and a mutation in *fre* (coding for a NAD(P)H flavin reductase); or harbored a mutation in *fusA* combined with a mutation in *yfgZ* (encoding a protein involved in repair during oxidative stress and Fe-S cluster synthesis). In general, in planktonic cultures clones were selected that had mutations in a diverse set of genes and MICs of these clones were typically lower than for clones evolved under biofilm conditions^104^. Interestingly, clones recovered from treated biofilms had higher survival rates upon treatment when grown in biofilms as compared to when grown in planktonic cultures, and the majority of evolved biofilm populations contained mutations in *fimH*, coding for the FimH tip-adhesin of type 1 fimbriae; the *fimH* mutants show enhanced biofilm formation and reduced amikacin susceptibility. Together these data suggest that the biofilm environment as such contributes to higher survival upon exposure to amikacin, by increasing the occurrence of new genetic resistance mutations, even in the absence of mutations that lead to increased tolerance^104^.

Experimental evolution of *Salmonella* Typhimurium biofilms grown on glass beads and planktonic cultures, in the presence and absence of azithromycin, cefotaxime and ciprofloxacin showed that biofilms and planktonic cultures develop resistance to these antibiotics in the same time frame^72^. However, the phenotype of evolved mutants differs between different conditions; e.g., in contrast to planktonic populations exposed to cefotaxime (which become mainly resistant to cefotaxime), biofilms evolved in the presence of cefotaxime show resistance to a wide range of antibiotics^72,127^. The same genes were often mutated in evolved planktonic and biofilm populations, e.g., mutations in *acrB* and *ramR* (after exposure to azithromycin), *envZ* (cefotaxime) and *gyrA* (ciprofloxacin); although the exact mutation sometimes differed (e.g., in *ramR:* term194Tyr in planktonic cultures vs. Thr18Pro in biofilms; in *gyrA*: Ser83Tyr in planktonic cultures vs. Ser83Phe in biofilms)^72,127^. These mutations suggest efflux (azithromycin), reduced membrane permeability (cefotaxime) and target modification (ciprofloxacin) are the most important mechanisms involved in the observed reduced susceptibility, although many other mutations were identified, and WGS clearly showed that different mutants followed different paths of adaptation.

### How does experimental evolution of biofilm susceptibility compare to evolution of susceptibility in vivo?

From the LTEE and many other studies we have learned that overall there is a high degree of parallelism in diversification and that evolution appears to be reproducible between replicate lineages and between different experiments carried out in different labs, suggesting the observed evolutionary changes are not random artefacts^15^. Additional proof for this comes from a direct comparison of mutations in experimentally evolved *P. aeruginosa* isolates and in clinical isolates, including those from chronic respiratory tract infections in CF. Overall these comparisons confirm that the changes observed in vitro are relevant for evolution of susceptibility in vivo. For example, selection of different ciprofloxacin resistance mechanisms is lifestyle-dependent^74^ which is in line with the high prevalence of mutations in ciprofloxacin target genes in isolates from acute infections (e.g., urinary tract infections), which are less common in isolates recovered from chronic infections^128^. Likewise, mutations in *P. aeruginosa* genes *fusA1* and *ptsP* occur in high frequency during in vitro evolution and identical mutations have been observed in clinical isolates^73,89^. *P. aeruginosa* adaptation to chronic infection not only occurs in CF; e.g., also in isolates recovered from chronic obstructive pulmonary disease patients mutations occur in genes that are frequently identified in experimental evolution studies (including *mexA, mexB, oprM* and *oprF*)^129^.

Indirect evidence comes from the comparison of phenotypes of isolates evolved in vitro with those involved during chronic infection^54^. For example, *P. aeruginosa* isolates recovered from younger CF patients typically display low resistance and low tolerance to antibiotics, and the frequency of drug-tolerant isolates increased with increasing age; increased frequencies of resistant isolates were only observed in older patients^58^. In these older patients two subpopulations were present, one consisting of highly resistant isolates and one consisting of hyper-tolerant isolates that retained low-level resistance, suggesting that also in vivo tolerance can be a ‘stepping stone’ towards resistance development^58^. Finally, the recent finding that biofilms are also present in at least some acute respiratory tract infections and that the main difference between acute and chronic infection may not be the association with the planktonic and biofilm lifestyle, respectively, but rather be related to differences in metabolism^130^ is in line with observations from in vitro experimental evolution studies as mutations in genes related to metabolism are frequently identified during experimental evolution^73,74,121^.

While these similarities between evolution in vitro and in vivo strongly suggest that genetic changes identified in vitro are relevant for what happens in vivo, experimental validation of the link between these genetic changes (in metabolism-related genes and others) on the one hand, and reduced antimicrobial susceptibility on the other, remains necessary.

### Changes in biofilm formation during experimental evolution

Changes in biofilm forming capacity during experimental evolution can also affect biofilm susceptibility. In the presence of daptomycin, *Enterococcus faecalis* biofilms grown in a bioreactor quickly develop resistance to the antibiotic, but at the same time biofilm formation increased in daptomycin-resistant strains^81^. WGS identified combinations of mutations that ultimately lead to an increase in biofilm formation and while this increase in biofilm formation is not a prerequisite for increased resistance, it was observed in the majority of the resistant lineages^81^. Increases in biofilm formation were also observed during experimental evolution of *A. baumannii* biofilms (both in the bead model^69^ and in a flow system^124^), with isolates from untreated and ciprofloxacin-treated biofilms showing increased biofilm formation capability compared to start cultures in both studies. Moreover, in the flow system, many isolates from tetracycline-treated biofilms showed an additional increase in biofilm formation^124^. While some mutations linked to increased biofilm formation occurred in treated and untreated samples (e.g., mutations in ABUW_0885 coding for biofilm-associated protein Bap), others (e.g., mutations in ABUW_2055, encoding a fimbrial adhesin) only occurred in untreated biofilms^124^. As already outlined above, *fimH* mutations were found in the majority of *E. coli* biofilm populations treated with amikacin as well as in the untreated controls; *fimH* mutants showed increased biofilm forming capacity and increased survival upon exposure to high concentrations of amikacin^104^. In studies with *Salmonella* Typhimurium biofilms grown on glass beads, a clear trade-off between antimicrobial resistance and biofilm formation was observed^72,127^. Over the course of the experiment, biofilm forming capacity (as measured by crystal violet staining) increased in colonies recovered from untreated glass beads and this was associated with a missense mutation in *cytR* (which is known to increase biofilm formation) that occurred in multiple untreated lineages^72^. However, colonies recovered from biofilms evolved in the presence of antibiotics (especially azithromycin and cefotaxime) showed reduced biofilm formation compared to unexposed biofilms and none of them contained mutations in *cytR*^72^. Exposure to subinhibitory concentrations of cefotaxime selects for mutations in the C-terminal catalytic/ATP-binding domain of EnvZ which result in lower levels of the porin OmpF and reduced permeability. However, EnvZ also regulates curli production and reduced curli production and biofilm formation was observed in *envZ* mutants, suggesting a trade-off between biofilm susceptibility and biofilm formation. Overall, these data suggest that the association between changes in biofilm formation and antimicrobial susceptibility during experimental evolution is complex and probably species, model and antibiotic-dependent.

## Looking at biofilm antimicrobial susceptibility through the lens of experimental evolution—a consensus view emerges

Although the studies discussed above used different model systems, antibiotics, species and strains, some common patterns emerge.

While decreased susceptibility during experimental evolution develops both in planktonic and biofilm populations, the mechanisms involved and the trajectories towards this reduced susceptibility are not identical. Mutations in genes that code for targets of antibiotics are frequently encountered in planktonic populations evolved in the presence of antibiotics (e.g., mutations in *gyrA* following evolution in the presence of ciprofloxacin), while evolved biofilm populations also contain a wide range of mutations in genes involved in efflux and metabolism^69,74,121^. When subinhibitory concentrations of antibiotics are used, growth in well-mixed planktonic cultures selected for high-level resistance, while growth in spatially structured biofilms favored mutants with lower levels of resistance^69,74,121^. However, this is not always the case when stepwise increasing or lethal concentrations of antibiotic are used during evolution^69,73,88,104^. While species- and/or antibiotic-dependent effects cannot yet be ruled out, this suggest that the treatment regime itself plays an important role in determining final MIC levels in planktonic and biofilm populations.

Evolved biofilm populations maintain a higher diversity than corresponding planktonic populations, in which successful mutations reach fixation quickly, and the biofilm environment may protect against negative selection of less fit resistant mutants that would be quickly outcompeted in planktonic cultures^121^. However, a recent study indicated that fitness costs for resistance in surface-associated *E. coli* biofilms did not differ from those in planktonic cultures^79^. In addition, another recent study has shown that the specific environment co-determines fitness and resistance levels associated with specific mutations^131^. Clearly more work is needed to gain deeper insight in parameters affecting fitness in different (structured) environments. In addition, mutations that lead to increased biofilm formation can increase the size of the tolerant population that survives antimicrobial exposure, in which resistance can subsequently develop^104^.

While mutations in some genes are found across organisms (e.g., mutations in *fusA* have been observed in *P. aeruginosa, A. baumannii* and *E. coli*), different organisms will also accumulate mutations in different genes although the resulting phenotype could be similar (Table 1). An example of such a parallel strategy are mutations in *P. aeruginosa orfKHLN* and *A. baumannii cyoAB*: while these genes are involved in very different cellular process (O antigen biosynthesis and electron transport, respectively) mutations in either result in reduced permeability for aminoglycosides and may lead to reduced aminoglycoside susceptibility^73^. Likewise, mutations in many different metabolic genes or sigma factors might lead to reduced growth, and ‘tolerance by lag’. This suggests that the fundamental mechanisms behind reduced biofilm susceptibility could be similar for different classes of antibiotics and in different organisms, even when it is not possible to identify mutations, mutated genes, or differences in metabolism or gene expression shared between different organisms. As such, data from experimental evolution are in line with the conclusion of a recent study that could not find evidence for a common genetic or biochemical basis for antimicrobial tolerance in biofilms but concluded that many genes, proteins, and metabolic pathways collectively determine the physiological state and susceptibility of bacterial cells in a biofilm^132^.

We believe experimental evolution has and will continue to help to elucidate the interplay of resistance, tolerance and persistence that is behind the reduced antimicrobial susceptibility of biofilms and determines the outcome of antimicrobial treatment. However, identifying the complex patterns of mutations, changes in gene expression and metabolism in different organisms as well as polymicrobial communities will require an interdisciplinary and holistic approach and will greatly benefit from the use of relevant model systems.

## Data Availability

Data sharing not applicable to this article as no datasets were generated or analysed during the current study.
